# Treatment of Canine Atlantoaxial Subluxation with a Modified Cervical Distraction–Stabilization Technique and Clinical Outcomes

**DOI:** 10.3390/ani15050716

**Published:** 2025-03-03

**Authors:** Giuseppe Barillaro, Marco Tabbì, Simone Minniti, Nicola Maria Iannelli, Francesco Macrì, Claudia Interlandi

**Affiliations:** 1 CVSG (Clinica Veterinaria San Giorgio), Via Vecchia Pentimele, 63, 89121 Reggio Calabria, RC, Italy; info@cvsg.it; 2 Department of Veterinary Sciences, University of Messina, Polo Universitario dell’Annunziata, 98168 Messina, ME, Italy; simoneminniti1993@gmail.com (S.M.); nicolamaria.iannelli@unime.it (N.M.I.); francesco.macri@unime.it (F.M.); cinterlandi@unime.it (C.I.)

**Keywords:** atlantoaxial instability, atlantoaxial subluxation, toy-breed dog, distraction–stabilization technique

## Abstract

Atlantoaxial subluxation (AAS) is a neurosurgical condition caused by joint hyperflexion and is characterized by the development of compressive cervical myelopathy. Various ventral fixation techniques have been described to permanently reduce the subluxation by long-term stabilization of the atlantoaxial joint. However, the rate of complications related to implant failure with these techniques remains high. The aim of this study was to describe a cervical distraction–stabilization technique using screws and PMMA to treat AAS and evaluate the clinical outcomes based on neurological and radiographic follow-up. Magnetic resonance imaging (MRI) was performed to confirm the diagnosis of AAS and identify any associated intraparenchymal lesions. Computed tomography (CT) scans were performed for surgical planning. No complications resulting in irreversible damage, surgical revision, or euthanasia protocol were observed. In the immediate postoperative period, the neurological scores showed an improvement of approximately two points from the preoperative values in all subjects. At the 6-month neurological examination (medium-term follow-up), 93% (13/14) of the dogs showed normal neurological function with no signs of pain. Based on clinical follow-up data, the cervical distraction–stabilization technique described in this study appears to be a valuable surgical alternative to treat AAS in toy-breed dogs.

## Simple Summary

This study describes a cervical distraction–stabilization technique using screws and PMMA to treat atlantoaxial subluxation (AAS) in 14 toy-breed dogs. Following surgery, an improvement in neurological status was documented in 13/14 cases. Based on clinical follow-up data, the cervical distraction–stabilization technique described in this study appears to be a valuable surgical alternative to treat AAS in toy-breed dogs.

## 1. Introduction

Atlantoaxial subluxation (AAS) is a neurosurgical condition of the cervical spine caused by atlantoaxial instability (AAI) [1,2]. This instability causes joint hyperflexion with dorsal subluxation of the axis relative to the atlas and the development of compressive cervical myelopathy [3]. In the acquired form, sudden subluxation usually causes spinal cord injury with acute or hyperacute onset. In contrast, in the congenital form, vertebral displacement and spinal cord compression are gradual, and the onset of neurological signs is insidious and progressive [4,5,6]. The congenital form is the most commonly diagnosed [7] and usually affects young dogs of small or toy breeds such as Yorkshire terriers, Chihuahuas, Poodles, Pomeranians, and Pekingese [1,2,8,9,10,11,12,13]. Agenesis, hypoplasia, and dysplasia of the dens or ligamentous supports, such as agenesis of the transverse ligament but also incomplete ossification of the atlas and blocked cervical vertebrae, are the main congenital or developmental lesions causing AAI [1,4,6,8,9,14,15,16,17,18,19,20,21,22].

Surgical treatment aims to permanently reduce the subluxation by long-term stabilization of the atlantoaxial joint [1,8,10]. However, long-term stabilization presents many challenges including bone immaturity, small size and narrow corridor for implant placement, and surgical proximity to vital structures. Various surgical techniques using a ventral approach for atlantoaxial stabilization have been described including the use of plates, screws, Kirschner nails, and wires, with or without polymethylmethacrylate (PMMA) [2,8,9,23,24,25,26,27,28,29,30,31,32,33,34]. However, various complications related to the surgical technique such as wheezing, coughing, laryngeal paralysis, dyspnea, dysphagia, pulmonary edema, pneumonia, or complications related to implant failure have also been reported with these techniques [2,6].

Given the variety of treatments proposed for this pathology and the complications associated with implants, the aim of this study was to describe a technique of cervical distraction–stabilization with screws and PMMA and report the outcomes and complications in 14 dogs with AAS. The technique described in this study is a modification of the technique originally described by Platt et al. [34]. The main difference from the original technique is the placement of two screws in the C1 body instead of three, the absence of transarticular Kirschner pins, and the orthopedic wire was wrapped around the head of the screws. Our aim was to use fewer implants to reduce the risk of complications while still providing adequate load distribution and stiffness.

## 2. Materials and Methods

### 2.1. Animals and Inclusion Criteria

Twenty-eight (n = 28) dogs with atlantoaxial subluxation (AAS) were surgically treated with a ventral cervical distraction–stabilization technique at the CVSG (Clinica Veterinaria San Giorgio) between 2018 and 2024.

The presumptive diagnosis of atlantoaxial instability was made in all subjects based on routine physical and neurological examinations. The diagnosis was then confirmed by radiography (RX) and magnetic resonance imaging (MRI) of the cervical spine and spinal cord. Before surgery, a peripheral blood sample was collected from all subjects for a complete blood count (CBC) and serum biochemistry (BUN blood urea nitrogen; CREA creatinine; BUN/CREA ratio; TP total protein; ALB albumin, GLOB globulin, ALB/GLOB ratio, GLU glucose, ALT alanine aminotransferase, ALKP alkaline phosphatase) to assess the health status and exclude the presence of comorbidities. Informed consent was obtained from the owner of each patient.

### 2.2. Diagnostic Imaging

**Figure 1:**
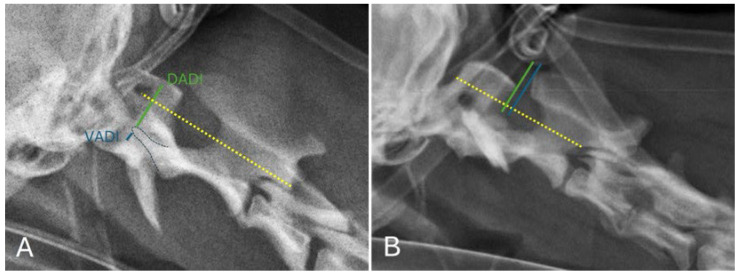
Neutral lateral radiographic projection of the atlantoaxial joint: ventral atlantodental interval (VADI) (blue line) and dorsal atlantodental interval (DADI) (green line) (**A**); line tangent to the caudal border of the dorsal arch of C1 (green line) and line tangent to the cranial border of the spinous process of C2 (blue line) (**B**).

Radiographs of the cervical spine were taken in latero-lateral projection, with the spine in a neutral position and parallel to the X-ray table, and with the cervical spine positioned directly under the primary X-ray beam. Three radiographic morphological indices were assessed on cervical radiographs: the ventral atlantodental interval (VADI), the dorsal atlantodental interval (DADI), and the C1–C2 overlap. The VADI was measured by drawing a line perpendicular to the longitudinal axis of the dens of C2, extending from the dorsal part of the ventral arch of C1 and ending at the ventral aspect of the dens of C2. The DADI was measured with a line perpendicular to the longitudinal axis of the dens but extending from the ventral part of the dorsal arch of C1 and ending at the dorsal aspect of the dens of C2. The ventral compression index (VCI) was calculated as the ratio of the ventral atlantodental interval (VADI) to the dorsal atlantodental interval (DADI) (VADI/DADI) (Figure 1A). The C1–C2 overlap was determined by measuring the distance between two lines, both perpendicular to the longitudinal axis of the dens of C2, the first tangent to the caudal border of the dorsal arch of C1, and the second tangent to the cranial border of the spinous process of C2 (Figure 1B).

The definitive diagnosis of AAS was confirmed by MRI (AIRIS Vento LT, 0.3 Tesla, Hitachi Medical Corporation, Tokyo, Japan), which also identified any intraparenchymal lesions associated with instability such as edema, hemorrhage or syringomyelia. Anesthesia was induced with propofol (Propovet Multidose 10 mg/mL, Zoetis S.r.l., Rome, Italy; 4 mg/kg IV) and maintained with isofluorane (IsoFlo, Zoetis Italia s.r.l., 20124 Milan, Italy). All dogs were placed in the sternal recumbency, with the neck at the isocenter of the coil and the forelimbs pointing backward and parallel to the chest. The MRI examination protocol included a T2-weighted series in the sagittal and transverse planes and a pre- and post-contrast T1-weighted series in two transverse planes for the C1–C2 tract. The T2-weighted (T2W) sequences had a slice thickness of 3.5 mm, a repetition time (TR) of 4000 ms, and an echo time (TE) of 120 ms. The T1-weighted (T1W) sequences had a slice thickness of 3.5 mm, a TR of 520 ms, and a TE of 20 ms. STIR sequences were also acquired in the sagittal and transverse planes of the lesion. All sequences were acquired in the neutral position of the patient’s neck.

A CT scan was performed for surgical planning using the same anesthetic protocol as for the MR. All dogs were placed in sternal recumbency with the head and neck in a neutral position and the forelimbs placed caudally and parallel to the chest wall. A multi-slice CT scan (GE Optima 520 16-slice, GE Healthcare S.r.l., Milan, Italy) with a tube voltage of 120 kVp, a tube current of 195 mAs, a slice thickness of 1.25 mm, and an increment of 0.5 mm was used. Multiplanar reconstruction and volumetric rendering were used for image evaluation. In addition, all dogs underwent a post-operative CT scan to assess the correct positioning of the implant in the vertebral bodies and possible invasion of the spinal canal.

### 2.3. Surgical Treatment

All dogs were premedicated with methadone (Semfortan 10 mg/mL, Eurovet Animal Health B.V., Bladel, The Netherlands; 0.2 mg/kg IM) and anesthesia was induced with propofol (Propovet Multidose 10 mg/mL, Zoetis S.r.l., Rome, Italy; 4 mg/kg IV). Maintenance of anesthesia was achieved with isofluorane (IsoFlo, Zoetis Italia S.r.l., 20124 Milan, Italy). Electrocardiography, heart and respiratory rates, end-tidal CO_2_, blood O_2_ saturation, and non-invasive blood pressure were continuously monitored during anesthesia. All dogs received intravenous antibiotic therapy with cefazolin (Cefazolin TEVA, Milan, Italy; 20 mg/kg) at the time of the induction of general anesthesia.

After induction, each patient underwent an extensive trichotomy of the areas of interest and was positioned in dorsal recumbency by slightly elevating the cervical region approximately 4 cm above the table using padded support, avoiding excessive extension. The thoracic limbs were placed caudally and secured to either side of the thorax. The right side of the dog was positioned toward the edge of the operating table to improve access for the lead surgeon during the surgical approach. The upper jaw was secured to the operating table with tape to maintain axial alignment and prevent movement during surgery. After trichotomy and positioning, surgical scrubbing was performed to prepare the surgical field. Both proximal humeri were included in the sterile field to allow the harvesting of an autologous cancellous bone graft.

A standard ventral median approach to the cervical spine was performed, specifically the ventral aspects of C1, C2, and the cranial aspect of C3. The area of surgical interest between the intermandibular space and the manubrium of the sternum was aseptically prepared with surgical scrub. A ventral median longitudinal incision was made from the caudal third of the mandible to approximately 5 cm caudal to the caudal border of the thyroid cartilage of the mid-cervical larynx. The incision was deepened through the various planes until the muscular plane was identified. The sternohyoid and sternocephalic muscles were incised longitudinally and retracted to expose the trachea. The right sternothyroid muscle was isolated and detached from its insertion at the thyroid process of the larynx, taking care to protect the thyroid gland during dissection. The trachea and esophagus were retracted laterally together with the major vessels and recurrent laryngeal nerves. To expose the longus colli muscles, the C1–C2 intervertebral space was located by palpation of the ventral processes and ventral tubercle of C1. The muscle fibers were lifted from the ventral arch of C1 and the body of C2 until the joints were exposed. This allowed direct ventral access to the first two cervical vertebrae, which were located by palpation of the caudal margins of the wings and the ventral prominence of C1.

**Figure 2:**
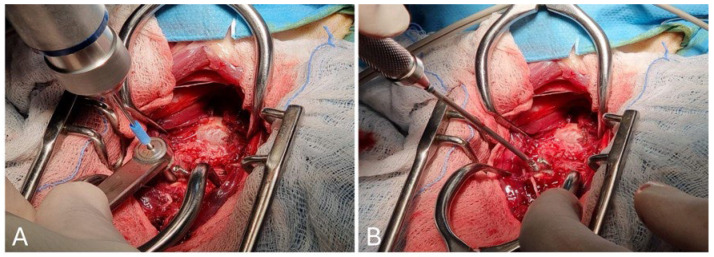
Predrilling with a 1.5 mm drill bit with guide: the blue band was positioned to ensure efficient locking based on the bone corridors and the selected screw size (**A**); screw insertion through the predrilled hole of a 2.0 mm self-tapping cortical locking screw (**B**).

To achieve proper alignment of the atlantoaxial joint and to protect the spinal cord during the procedure, two 1.5 mm or 2.0 mm self-tapping cortical bone screws were inserted into both the C1 and C2 bodies. Predrilling with a drill bit and guide was performed prior to screw insertion. A stop band was placed on the drill bit to ensure adequate predrilling depth. The screws were then inserted through the predrilled hole (Figure 2).

The first two cortical bone screws were inserted into the C1 body: one near the right wing and the other near the left wing. Two additional cortical bone screws were then inserted into the body of C2, one in the caudal half and one in the cranial half, both along the midline at the point of maximum thickness. The appropriate screw size and length was determined from radiographs and CT scans during surgical planning so that the thread would pass through both cortical bone layers without compressing the spinal cord, leaving the entire screw head and a short segment (2–3 turns) of the threaded portion exposed in the vertebral bodies. Overall, approximately 2/3 of the screw passed from the outer to the inner cortex, and 1/3 was left exposed to allow good PMMA grip. After the screw placement, a suture was twisted around the head of both C2 screws and caudoventral traction was applied and maintained. This manual vertebral distraction allowed for an indirect reduction in the atlantoaxial dislocation and better exposure of the facet joints.

After exposure of the C1–C2 facet joints by capsulotomy, chondrectomy was performed with a high-speed burr until complete exposure of the subchondral bone. An autologous bone graft was harvested from the greater tubercle of the humerus and placed in the joint space. The screw head sheaths were then filled with bone wax. At this point, the surgeon applied polymethylmethacrylate (PMMA) (CMW 1. DePuy Synthes) to the screw heads and bone surface while the assistant maintained the caudoventral traction to hold the cranial aspect of the C2 body stationary, thus maintaining C1–C2 distraction until complete solidification. The high viscosity, medium setting PMMA was molded to ensure complete adhesion to the screws and as little bulk as possible to ensure normal tissue apposition, and then rinsed copiously with saline at room temperature until the exothermic reaction had completely disappeared. Muscles were repositioned on the implants where possible, and the remainder of the wound closure was performed routinely. The average duration of the procedure was 75 min (range: 60–90 min). A rigid cervical brace was applied to all patients.

### 2.4. Postoperative Care

During the first 24 h after surgery, each patient received antibiotic treatment with cefazolin (Cefazolin TEVA, Milan, Italy; 20 mg/kg, IV q12h) and analgesic treatment with methadone (Semfortan Eurovet Animal Health B.V., Handelsweg, The Netherlands; 0.2 mg/kg, IM, q6h). Both antibiotic and analgesic treatment were then replaced by the oral administration of cefadroxil (Cefa-Cure Tabs cpr, MSD Animal Health S.r.l., Milan, Italy; 20 mg/kg, q24h for 7 days) and tramadol (Altadol, Formevet S.r.l., Milan, Italy; 2–4 mg/kg, q8h), respectively. Postoperative analgesia was adapted to the clinical course and needs of each patient. In addition, oral anti-inflammatory therapy with meloxicam (Metacam, Boehringer Ingelheim Animal Health Italy S.p.A., Noventana, Italy; 0.2 mg/kg on the first day, then 0.1 mg/kg, q24h for 10 days) and gastroprotective therapy (omeprazole at 1 mg/kg q24h and sucralfate at 50 mg/kg q8h, both OS for 15 days) were administered. Finally, oral supplementation with palmitoylethanolamide (PEA) and quercetin (Alevica^®^, Innovet, Saccolongo-Padua, Italy; q24h for at least 20 days) was prescribed.

Owners were instructed to follow clear and strict instructions during the postoperative period. These included keeping the patient in a confined space, avoiding sudden exertion or jumping from any height, and walking as little as possible, for short periods, and always with a support sling. After 6 weeks, the brace was removed, and follow-up X-rays of the cervical spine were taken. If the follow-up radiographs showed no signs of complications (screw brakeage or implant dislocation), a gradual increase in the frequency and duration of walking was allowed for a further 6 weeks before full activity was resumed.

### 2.5. Follow-Up

Each dog underwent clinical, neurological, and radiographic examinations before and immediately after surgery and at 6 weeks after surgery (short-term follow-up). Short-term neurological status was compared before and after surgery and dogs were classified as normal, improved with residual dysfunction, unchanged, or worse. Neurological signs were graded according to a modified Frankel scale previously described [35].

Postoperative radiographs were taken in both standard orthogonal projections to assess implant positioning and integrity. Radiographs were taken under sedation to achieve optimal patient positioning. Clinical and neurological assessments were also performed 6 months after surgery (medium-term follow-up).

Complications and recurrence of clinical signs were recorded. Complications were classified as catastrophic (permanent unacceptable function directly related to death or euthanasia), major (additional treatment required), or minor (additional treatment not required) [36]. In addition, complications were categorized as intraoperative, short-term (6 weeks after surgery) and medium-term (6 months after surgery).

## 3. Results

### 3.1. Animals

Twenty-eight (n = 28) dogs affected by atlantoaxial subluxation (AAS) were surgically treated. Subjects with concomitant systemic diseases (n = 6) or those who did not comply with the planned postoperative follow-up (n = 8) were excluded from the study. Fourteen (n = 14) owned dogs were included in the study. The study group consisted of eight males (57%) and six females (43%) aged between 2 and 3 years (Table 1). Weight ranged from 1.5 kg to 4.1 kg, with a mean of 2.76 ± 0.8. The dogs were fed commercial diets and were housed indoors and outdoors.

### 3.2. Preoperative Assessment

During the anamnesis, the most common clinical signs reported by the owners were neck stiffness with ventroflexion and neck pain on manipulation, reluctance to move, and hind limb weakness that gradually worsened and spread to the forelimbs with proprioceptive ataxia. General clinical examination revealed atrophy of the supra- and infraspinatus muscles in all patients.

On neurological examination, the most common clinical signs were neck pain and stiffness with ataxia, gait tetraparesis with short, stiff forelimb gait and dysmetric hindlimb gait (so-called “two-engine” gait), and varying degrees of proprioceptive deficits (14/14, 100%), sometimes severe (7/14, 50%), localized to all limbs with hyperreflexia of the limb flexor reflex. In the most severe cases, non-ambulatory tetraparesis was observed (1/14, 7%). No subject was found to be tetraplegic. All patients had normal cranial nerve examinations.

The radiographs of all subjects showed a dorsal displacement of the dens of C2 within the spinal canal. Evaluation of the three objective radiographic morphological indices (VADI, DADI, C1–C2 overlap) and VCI (VADI/DADI ratio) showed an increased VCI (mean value 0.42 ± 0.11; median 0.380) and a decreased C1–C2 overlap (mean value −2.69 ± 1.31; median −3.320).

### 3.3. Postoperative Evaluation

In all subjects included in the study, there was an improvement in neurological scores after surgical treatment. Immediately after surgery, the neurological scores improved by approximately two points on the neurological scale used in all subjects. At 6 weeks after surgery (short-term follow-up), a further improvement of approximately one point on the neurological scale was observed in subjects compared with the immediate postoperative period. In 50% (7/14) of the subjects, a neurological score of 0 was already observed at the short-term follow-up. At 6 months after surgery (medium-term follow-up), 93% (13/14) of the dogs showed normal neurological function with no signs of pain, while only 7% (1/14) still showed signs of neck pain. The neurological scores of the subjects are shown in Table 2.

**Figure 3:**
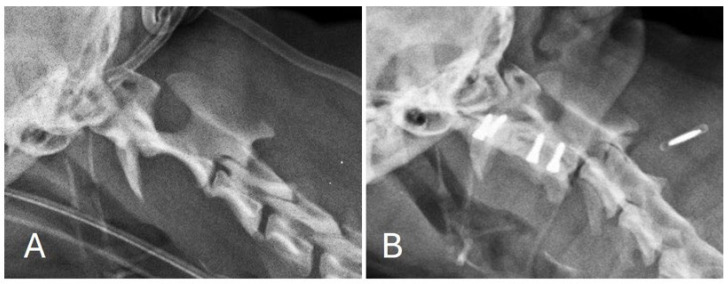
Lateral view of the cervical spine before (**A**) and after (**B**) surgical treatment. Correct screw insertion can also be observed.

**Figure 4:**
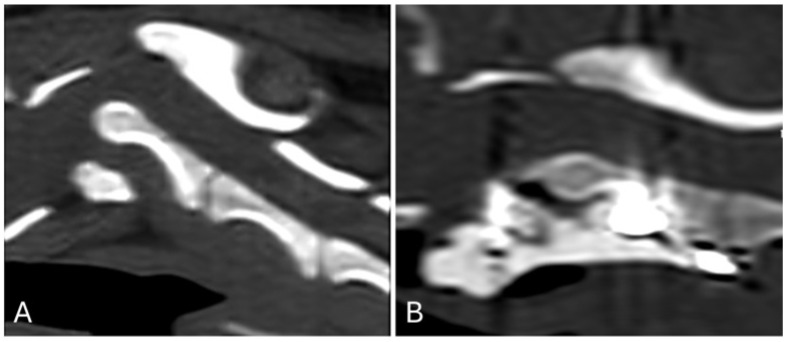
Sagittally reconstructed pre-operative (**A**) and post-operative (**B**) CT images of the atlantoaxial region.

Post-operative images showed correct implant positioning and normal location of the dens within the respective fovea along the ventral arch of C1 (Figure 3 and Figure 4).

No catastrophic, major, or minor complications were observed in the post-operative images. No surgical revision or euthanasia protocol was required.

## 4. Discussion

The main advantage of the cervical distraction–stabilization technique described in this study is the reduced risk of iatrogenic damage due to the absence of transarticular Kirschner pins and orthopedic wire. Adequate load distribution and stiffness were provided by the placement of cortical screws and PMMA.

Atlantoaxial subluxation is a neurosurgical condition of the cervical spine that leads to compressive cervical myelopathy [3]. Congenital SAA is the most common form [7] and predominantly affects young dogs aged ≤4 years old, small, or toy breeds [1,2,9,10,11,13]. All subjects included in our study were small dogs (4/14, 28%) or toy breeds (10/14, 72%), aged between 2 and 3 years with body weights ranging from 1.5 to 4.1 kg. None of the subjects included in the study were younger than 24 months of age, which has been reported to be a positive predictor of SAA stabilization success [2]. A comparative statistical evaluation of patient age as a predictive factor was not possible in our study due to the small sample size.

The clinical signs of SAA are typical of a C1–C5 compressive myelopathy. Therefore, neurolocalization is essential to exclude other possible pathologies. On neurological examination, the most common clinical signs were cervical pain and stiffness with ataxia, ambulatory tetraparesis with a short, stiff gait of the forelimbs and a dysmetric gait of the hindlimbs (so-called “two-engine” gait), and varying degrees of proprioceptive deficits (14/14, 100%), sometimes severe (7/14, 50%), extending to all limbs with limb flexor reflex hyperreflexia. In the most severe cases, non-ambulatory tetraparesis was observed (1/14, 7%). The clinical presentation observed was consistent with that previously reported in the literature.

However, confirmation of the diagnosis of SAA requires the use of imaging techniques such as radiography, computed tomography (CT), and magnetic resonance imaging (MRI). Radiography is the primary diagnostic technique due to its accessibility and widespread availability in veterinary practice. Several objective radiographic measurements have been described in veterinary medicine to support the diagnosis of IAA. Among these, the VCI (VADI/DADI ratio) is a relative measure that allows a measurement of high specificity independent of body weight, while the C1–C2 overlap showed the highest combination of sensitivity and specificity but was significantly related to body weight [37]. In our study, the radiographs of all subjects showed a dorsal displacement of the dens of C2 within the spinal canal. Evaluation of the three objective radiographic morphological indices (VADI, DADI, C1–C2 overlap) and calculation of the VCI showed an increased VCI (mean value 0.42 ± 0.11; median 0.380) and a decreased C1–C2 overlap (mean value −2.69 ± 1.31; median −3.320). Although the median values of the radiographic measurements were highly indicative of atlantoaxial instability, both were replicated in CT. Furthermore, CT and MRI were used to obtain a more detailed assessment of bony anatomy and ligamentous support, identify any concomitant intramedullary lesions, and for surgical planning.

Management of IAA includes both medical and surgical treatment. Medical treatment generally involves immobilization of the joint with a cervical splint, cast, or brace for approximately 6 weeks [10,16], together with drug therapy for pain management and strict exercise restriction [38]. Prolonged use of a cervical brace can lead to various complications such as dermatitis, external otitis, corneal, and skin ulcers. In addition, the lack of true permanent joint fusion is associated with a high risk of recurrence [10,38]. For these reasons, conservative management is reserved for patients with mild neurological signs or mild cervical hyperesthesia, or in cases where the surgical approach is limited (immature skeleton, high anesthetic risk, or economic constraints of the owner) [6,10,25].

Surgical treatment is indicated in most cases and in all patients with severe neurological symptoms and severe neck pain, or in cases refractory to medical management [1,6,10,25]. The various surgical approaches described for the treatment of AA are generally divided into dorsal and ventral fixation techniques [6,21,22,38].

The dorsal approach avoids vascular and nerve structures and was therefore considered technically simpler and safer than the ventral approach (Forterre). However, it does not allow arthrodesis by chondrectomy of the articular surfaces as it does not provide direct access to the atlantoaxial joint [2,39].

Furthermore, dorsal stabilization techniques only resist loading in flexion and not rotation and shear. Consequently, such joint movements may delay the formation of fibrous scar tissue and increase the risk of early failure and recurrence. Complications of dorsal procedures include implant failure or rupture with loss of reduction, fracture of C1 and C2, severe spinal cord damage with cardiorespiratory arrest, and death [2]. The ventral approach is now the preferred technique for treating SAA because it provides greater biomechanical stability by allowing the surgical implant to be fixed directly to the ventral surface of the first two cervical vertebrae. The complete exposure of the vertebral bodies and articular surfaces allows for true arthrodesis, promoting complete bone fusion between C1 and C2. Although the need for ankylosis of the joint for long-term stabilization has not been demonstrated, bone fusion may be beneficial, as in other cervical spine conditions requiring ventral distraction stabilization techniques [23,28,35]. However, previous studies have confirmed the presence of varying degrees of bone union at the atlantoaxial joint in dogs treated with ventral stabilization techniques as early as 6 weeks after surgery [27]. The actual arthrodesis is assessed by radiography under anesthesia, although this is difficult due to the use of PMMA [28].

One of the main challenges in the surgical stabilization of AAS is the anatomy of the first two cervical vertebrae. Toy-breed dogs, which are typically affected, frequently have immature bones and thin, fragile cortices [38]. The high fragility of these structures and the extremely limited bone surface available for implant placement contrasts with the need to ensure good implant rigidity. An optimal stabilization must be able to absorb, distribute, and dissipate all forces exerted on the joint. For this reason, most atlantoaxial stabilization techniques aim to maximize both the number and size of the implants used.

The most widely used ventral techniques use Kirschner wires or nails to increase implant stiffness. However, misplacement, migration, or rupture of the Kirschner wires or nails, with loss of reduction in the atlantoaxial joint, or, in the worst cases, spinal canal injury and death remain the most reported complications [2,3]. Nail migration is associated with fracture, probably due to residual joint motion that promotes cyclic bending of the nails until they break or due to damage of the nail during its insertion.

The ventral technique proposed in our study aimed to achieve the same level of stability and rigidity as other more complex approaches, but using only 1.5 mm or 2.0 mm self-tapping cortical screws and high viscosity, medium setting PMMA bone cement.

The decision not to use Kirschner wires or nails, either transarticularly or by cerclage, allowed us to reduce the risk of iatrogenic damage by isolating this possibility only at the stage of screw placement, while at the same time reducing the duration of the operation. However, the absence of these elements did not reduce the rigidity of the implant, which was guaranteed by the placement of four cortical screws and the use of PMMA.

Transarticular screw fixation (TSF) and cemented polymethylmethacrylate (PMMA) constructs have been the most widely used ventral techniques in recent decades with high success rates (50–94%) [2,8,9,13,17,24,25,26,27,28,29,30,31,32]. Although the C2 pedicle screw in TSFs can result in excellent implant positioning without spinal canal injury, these positions almost inevitably result in vertebral artery injury, which is still of unknown clinical significance in dogs. A recent study showed that the bone corridor available for TSF screw placement in toy dogs varies from 3 to 4.5 mm in diameter, with a maximum of 3.5 mm in the narrowest areas, making such placement risky in toy breeds, even with proper preoperative planning [40]. The midline screw positioning described in this study allows for a better distribution of forces along the C2 vertebral body, thus eliminating the fulcrum effect generated by TSF screws, which frequently causes the failure of TSF constructs secondary to a bone fracture located at the C2 cranial articular surface. Individualized pre-operative planning with precise length measurements of the bone corridor and drill stop systems allowed for any complications related to screw positioning, such as spinal canal violation and vertebral artery injury, to be avoided.

One of the advantages of PMMA constructs is their biomechanical superiority compared with TSF. According to some biomechanical studies, PMMA constructs would resist implant failure due to an acute sudden increase in ventral bending loads better than TSFs, which are more prone to failure [33,34]. The disadvantages of using PMMA include thermal damage, pressure necrosis of the cervical structures, and infection [2]. However, none of these complications were observed in the subjects included in the present study, probably due to adequate preoperative planning, which allowed us to calculate the exact size and choice of number of screws used, thus reducing the amount of PMMA applied. There were no difficulties or complications in performing our surgical technique. In fact, in all subjects included in the study, a reduction of no less than two points on the neurological scale was observed in the immediate postoperative period compared with the preoperative values. A further reduction of one point on the neurological scale was observed in the short-term follow-up, during which half (7/14, 50%) of the treated subjects showed complete recovery from SAA. Finally, at the medium-term follow-up, 93% of the subjects (13/14) showed normal neurological function, and only 7% (1/14) retained signs of mild neck pain. Based on the criteria used to define surgical success, which is an improvement in neurological status without recurrence or the need for further intervention, our technique was effective in all cases.

## 5. Conclusions

The conclusions of our study highlight the effectiveness of the ventral stabilization technique proposed for the treatment of atlantoaxial instability in toy dogs. The clinical results, supported by a neurological improvement in all treated subjects, confirmed the validity of this surgical approach, which offered a reduction in complications compared with traditional techniques. The exclusive use of self-tapping cortical screws and PMMA provided adequate implant rigidity and stability, minimizing the risks associated with previous techniques. In addition, the reduction in operating time and the absence of major or catastrophic post-operative complications further demonstrated the efficacy and safety of the surgical technique.

## Figures and Tables

**Table 1:** The table shows the breed, weight (kg), and age (years) of the subjects included in the study.

Subject	Breed	Weight (Kg)	Age (Years)
1	Chihuahua toy	1.5	2
2	Chihuahua toy	2.1	2.5
3	Chihuahua toy	1.7	3
4	Chihuahua toy	2.3	2
5	Chihuahua toy	1.9	3
6	Chihuahua toy	2.4	2.5
7	Poodle toy	3.8	2.5
8	Poodle toy	3.5	3
9	Poodle toy	4.1	2
10	Poodle toy	3.9	2.5
11	Pomerania	2	3
12	Pomerania	2.9	2
13	Maltese	3.2	2.5
14	Maltese	3.4	2

**Table 2:** Neurological scores of subjects included in the study at pre-operative (Pre-Op), immediate post-operative (Post-Op), short-term follow-up (FU-ST), and mid-term follow-up (FU-MT).

		Neurological Score			
Subject	Breed	Pre-Op	Post-Op	FU-ST	FU-MT
1	Chihuahua toy	3	1	0	0
2	Chihuahua toy	3	1	0	0
3	Chihuahua toy	4	2	1	0
4	Chihuahua toy	5	3	2	1
5	Chihuahua toy	4	2	1	0
6	Chihuahua toy	4	2	1	0
7	Poodle toy	3	1	0	0
8	Poodle toy	4	2	1	0
9	Poodle toy	4	2	1	0
10	Poodle toy	3	1	0	0
11	Pomeranian	3	1	0	0
12	Pomeranian	3	1	0	0
13	Maltese	4	2	1	0
14	Maltese	4	2	0	0

## Data Availability

The original contributions presented in this study are included in the article. Further inquiries can be directed to the corresponding author.
